# Preventive Effect of Aspirin Eugenol Ester on Thrombosis in κ-Carrageenan-Induced Rat Tail Thrombosis Model

**DOI:** 10.1371/journal.pone.0133125

**Published:** 2015-07-20

**Authors:** Ning Ma, Xi-Wang Liu, Ya-Jun Yang, Jian-Yong Li, Isam Mohamed, Guang-Rong Liu, Ji-Yu Zhang

**Affiliations:** Key Lab of New Animal Drug Project of Gansu Province, Key Lab of Veterinary Pharmaceutical Development, Ministry of Agriculture, Lanzhou Institute of Husbandry and Pharmaceutical Science of CAAS, Lanzhou, 730050, P. R. China; Faculty of Pharmacy, Ain Shams University, EGYPT

## Abstract

Based on the prodrug principle, aspirin eugenol ester (AEE) was synthesized, which can reduce the side effects of aspirin and eugenol. As a good candidate for new antithrombotic and anti-inflammatory medicine, it is essential to evaluate its preventive effect on thrombosis. Preventive effect of AEE was investigated in κ-carrageenan-induced rat tail thrombosis model. AEE suspension liquids were prepared in 0.5% sodium carboxymethyl cellulose (CMC-Na). AEE was administrated at the dosage of 18, 36 and 72 mg/kg. Aspirin (20 mg/kg), eugenol (18 mg/kg) and 0.5% CMC-Na (30 mg/kg) were used as control drug. In order to compare the effects between AEE and its precursor, integration of aspirin and eugenol group (molar ratio 1:1) was also designed in the experiment. After drugs were administrated intragastrically for seven days, each rat was injected intraperitoneally with 20 mg/kg BW κ-carrageen dissolved in physiological saline to induce thrombosis. The length of tail-thrombosis was measured at 24 and 48 hours. The blank group just was given physiological saline for seven days without κ-carrageenan administrated. The results indicated that AEE significantly not only reduced the average length of thrombus, PT values and FIB concentration, but also reduced the red blood cell (RBC), hemoglobin (HGB), hematocrit (HCT) and platelet (PLT). The effects of AEE on platelet aggregation and anticoagulant *in vitro* showed that AEE could inhibit adenosine diphosphate (ADP)-induced platelet aggregation as dose-dependence but no notable effect on blood clotting. From these results, it was concluded that AEE possessed positive effect on thrombosis prevention *in vivo* through the reduction of FIB, PLT, inhibition of platelet aggregation and the change of TT and PT values.

## Introduction

Since aspirin was invented, its use has become wide spread in the prevention and treatment of inflammation, headache, fever and cardiovascular disease [1–6]. Eugenol, the principle chemical component of clove oil extracted from dry alabastrum of *Eugenia Caryophyllata Thumb*, has been recognized as safe essential oil by Food and Chemical Administration. Eugenol has been known for its various therapeutic effects, including anticoagulation, antivirus, analgesia, antipyretic, anti-inflammation, antibacterial, anti-platelet aggregation, antioxidation, anti-diarrhea, anti-hypoxia, antiulcer, and inhibition of intestinal movement and arachidonic acid metabolism [7–11].

However, the adverse reactions of aspirin such as gastrointestinal damage, have limited the long-term use of this classical drug [12]. Eugenol, which containing phenolic hydroxyl group, is irritant and vulnerable to oxidation. In order to reduce their side effect and improve therapeutic effect and stabilization, aspirin eugenol ester (AEE) was synthesized [13]. The acute toxicity, teratogenicity, metabolism, pharmacodynamics, stability and mutagenicity of AEE have been investigated. The acute toxicity of AEE indicated that the toxicity of AEE is less than that of aspirin and eugenol, only 0.02 times the toxicity of aspirin and 0.27 times the toxicity of eugenol in mice [13]. A 15-day oral dose toxicity study showed that no-observed-adverse-effect level (NOAEL) value of AEE was considered to be 50 mg/kg/day under the study conditions [14]. In the Ames test and the mouse bone marrow micronucleus assay, the AEE did not show any mutagenesis [15]. Metabolites of AEE *in vivo* and *in vitro* also have been confirmed by HPLC-MS/MS, which indicated that AEE was decomposed into salicylic acid and eugenol [16]. Moreover, the evaluation for its anti-inflammatory, analgesic and antipyretic effects through animal model showed that AEE possessed similarity effects with aspirin, but lasted for a longer time [17–18].

Cardiovascular diseases including hypertension, coronary heart disease, atherosclerosis and acute myocardial infarction are the major cause of morbidity and mortality. Intravascular thrombosis is one of the main causes in multiple cardiovascular diseases [19–20]. The physiological process of thrombosis is very complicated, which influenced by many factors such as blood vessel injury, blood flow, platelet adhesion and aggregation. Thrombosis starts with the injury of blood vessel, and then platelets and fibrin are used to form a blood clot. Even when blood vessel is not injured, blood clots may form in the body under certain conditions [21–22]. Thrombus formation will lead ischemic necrosis, which is a key factor for infarction. Inflammation and thrombosis were also essential pathogenetic factors in the initiation of atherosclerosis. Because of the key role of thrombosis in cardiovascular diseases, thrombosis should be responsible for much morbidity and mortality [23]. However, owing to the side effects and inconvenience of currently available agents, new antithrombotic and thrombolytic agents are still needed.

The purpose of this study was to investigate the preventive effect of AEE on κ-carrageenan-induced rat tail thrombosis model. The κ-carrageenan induced tail thrombosis model in rat is useful for evaluating whether compounds have antithrombotic effects in drug discovery stage [24–26]. The researchers could use this model to observe the progression of thrombosis visually and directly in a time-dependent manner. Moreover, this model is simple and no-invasive on lab animals. Based on these advantages, this model was chosen in the experiment. Hematological analysis and blood coagulation parameters examination including thrombin time (TT), prothrombin time (PT) and fibrinogen (FIB), were also carried out to evaluate the influence of AEE on animal thrombosis model.

## Materials and Methods

### Chemicals and reagents

AEE, transparent crystal (purity: 99.5% with RE-HPLC), was prepared in Key Lab of New Animal Drug Project of Gansu Province, Key Lab of Veterinary Pharmaceutical Development of Agricultural Ministry, Lanzhou Institute of Husbandry and Pharmaceutical Sciences of CAAS. CMC-Na was supplied by Tianjin Chemical Reagent Company (Tianjin, China). Aspirin and Tween-80 were obtained from Aladdin Industrial Corporation (Shanghai China). Eugenol was supplied by Sinopharm Chemical Reagent Co., Ltd. (Shanghai China). ADP and κ-carrageenan was purchased from Sigma (St. Louis, USA). Prothrombin time (PT) reagent, thrombin time (TT) reagent and fibrinogen (FIB) reagent were obtained from Sysmex Corporation (Fukuoka, Japan). All chemical reagents were of analytical reagent grade.

### Animals

Ninety Wistar male rats with clean grade, aged 7 weeks and weighing 150~160 g, were purchased from animal breeding facilities of Lanzhou Army General Hospital (Lanzhou, China). Animals were housed in stainless steel cages in a ventilated room. Light/dark regime was 12/12 h and living temperature is (22 ± 2)°C with relative humidity of (55 ± 10) %. Standard compressed rat feed from Beijing Keao Xieli Co., Ltd (Beijing China) and drinking water were supplied ad libitum. The study was performed in compliance with the Guidelines for the care and use of laboratory animals as described in the US National Institutes of Health and approved by Institutional Animal Care and Use Committee of Lanzhou Institute of Husbandry and Pharmaceutical Science of CAAS. Animals were allowed a 2-week quarantine and acclimation period prior to start of the study.

### Drug preparation

AEE and aspirin suspension liquids were prepared in 0.5% of CMC-Na. Eugenol and Tween-80 at the mass ratio of 1:2 mixed with the distilled water. κ-carrageenan was dissolved in physiological saline.

### Dose

Total of ninety rats were randomly divided into following groups (n = 10). Group 1 was served as blank group received physiological saline. Group 2 was served as animal disease model. Group 3 was given 5% CMC-Na 30 mg/kg as negative control. Group 4 was received 20 mg/kg aspirin as positive group. The high-, medium-, and low-doses of AEE were selected as 72, 36 and 18 mg/kg, organized into groups 5 to 7 respectively. Group 8 was given eugenol at the dosage of 18 mg/kg. Group 9 was received both aspirin and eugenol (aspirin: 20 mg/kg, eugenol: 18 mg/kg). After different drugs were administrated intragastrically for seven days, the rats in groups 2 to 9 were intraperitoneally injected with κ-carrageenan to induce thrombosis.

### Carrageenan-induced rat tail thrombosis model

Based on animal welfare, rationality and simplity of thrombosis model, κ-carrageenan was used to induce rat tail thrombosis for investigating preventive effect of AEE. The apparent formation character of rat tail thrombosis was swelling and redness which were observed from the experiment. For optimal model, κ-carrageenan dose and environment temperature, infarction time were tested and selected in the preliminary study. Environment temperature of 20°C was selected for the establishment of thrombosis model. The dose of κ-carrageenan at 20 mg/kg BW can guarantee all the carrageenan treated group to appear thrombus and have a suitable thrombus length in the tail. There was a little infarction in the tip end of rat tail after carrageenan injection at 4 hours, and then redness and swell were observed in the tail. The length of infraction was increased with the time elapsed and be stable at 24 hours after κ-carrageenan injection. Therefore, 24 hours was time point which thrombosis model was established successfully.

In the experiment, at half an hour from the last treatment with different drugs, each rat was intraperitoneally injected with 20 mg/kg BW κ-carrageenan dissolved in physiological saline. Rats were observed for the formation of thrombosis, swelling and redness at 20°C environment temperature. Thrombus lengths were measured and photographed at 24 and 48 h.

### Hematological analysis and coagulation parameters examination

In order to compare the effects between AEE and its precursor, aspirin group, eugenol group and integration of aspirin and eugenol (molar ratio 1:1) group were designed as control groups in the experiment, same molar quantity with medium-dose of AEE.

After drugs were administrated intragastrically for seven days, thrombosis disease model was induced by carrageenan, and then the length of tail thrombosis was measured and photographed at 24 and 48 h. After the last measurement of thrombosis length, rats were anesthetized with 10% chloral hydrate and blood samples were taken from heart for hematological analysis and blood coagulation parameters examination.

2 ml blood samples were collected into EDTA-K_2_ vacuum tubes for hematological analysis and measured on a Sysmex XE-2100 analyzer. Same amount of blood samples were collected and diluted by 3.2% sodium citrate on the proportion of 9:1 in vacuum tubes, then serum be got after centrifuging for 15 minutes in 3000 rpm. The serum was measured for blood coagulation parameters examination on Sysmex CS-5100.

### Assay of anticoagulant effect of AEE *in vitro*

The method applied to anticoagulant assay was according to the related reference and made little modification [27]. 1 ml fresh rat blood samples were collected, and different doses of AEE were added into these samples. In order to solve the problem of indissolvableness of AEE, dimethyl sulfoxide (DMSO) was used as vehicle. The control was added with 200 μL DMSO in 1 ml blood. The final concentration of AEE in blood samples were 1 μg/ml, 10 μg/ml, 100 μg/ml and 200 μg/ml, respectively. After mixed gently, the mixture was incubated at 37°C for 30 min. Then the anticoagulant effects of AEE on rat fresh blood were observed.

### Assay of platelet aggregation *in vitro*

The *in vitro* activity studies on anti-platelet aggregation of AEE have been done by using Born test [28]. Rat blood samples gathering from the rat heart were collected into vacuum tubes which contained 3.8% sodium citrate (1:9, v/v). Platelet aggregation was assessed in platelet-rich plasma (PRP) obtained by centrifugation of citrated whole blood at room temperature for 10 min at 1000 rpm. The aggregation rate was measured by platelet aggregation analyzer (Chrono-log Model: 700) after stimulation with ADP (5 μM) using platelet-poor plasma (PPP) to set zero. The PPP was obtained by centrifugation of PRP at room temperature for 10 min at 3000 rpm.

The solution of AEE and aspirin dissolved in DMSO (5 μL) was added into PRP (250 μL), and the same volume of DMSO with no test compound was added as a reference sample. After incubation of rat platelets with AEE at concentrations ranging from 50 μg/ml to 200 μg/ml for 10 minutes, the platelet aggregation activities was assessed using ADP (5 μM). In the experiment, aspirin was used as positive control drug. Results were recorded at maximal aggregation after the addition of ADP. Data are expressed as percentage of maximal aggregation.

### Statistical analysis

The statistical analyses were carried out using SAS 9.2 (Statistics Analysis System USA). Data obtained from experiment was expressed as mean ± standard deviation (SD). Statistical differences between the treatments and the control were evaluated by one-way ANOVA. *P*-value less than 0.05 were considered to indicate statistical significance.

## Results

### Effects of AEE on rat thrombosis model

The full-length of rat tail was measured in the experiment, and then the data was analyzed by Student’s t-test. The results showed that there was no difference in the tail full-length. After 4 hours of κ-carrageenan treatment, swelling and redness were observed in model group, while swelling and redness did not occur in the group which was given AEE at the dosage of 72 mg/kg. The lengths of thrombosis at 24 and 48 hours were shown in Table 1. There were not significant differences in aspirin group when compared with CMC-Na group at 24 and 48 hours. However, there were significant differences in eugenol and AEE groups at the time interval (24–48h) (*P*<0.01 in eugenol and AEE groups), which showed that eugenol and AEE possessed obvious antithrombotic effect in this thrombosis model. From 24 to 48 hours, the thrombosis lengths in model group were increased but other groups remained unchanged. There was no difference between model group and CMC-Na group in thrombosis length at 48 hours, which indicated that CMC-Na had no influence on thrombosis formation at 48 hours. The average thrombus lengths at 48 hours in low-dose and medium-dose AEE group were 9.63 cm and 8.96 cm, respectively, which showed the stronger antithrombotic effect of AEE with the amount increased. However, the average lengths between medium-dose and high-dose AEE groups were not significant different.

**Table 1:** Preventive effects of AEE in κ-carrageenan-induced rat tail thrombosis model (n = 10). Note: After different drugs were administrated intragastrically for seven days, rat tail thrombosis model was induced by κ-carrageenan at a dose of 20 mg/kg BW, and then the length of tail thrombosis was measured at 24 and 48 h. Values are presented as mean ± SD (n = 10).

Groups	Dose (mg/kg)	Tail full-length (cm)	Thrombosis length (cm)	
			24 Hours	48 Hours
Model	—	15.88±0.38	11.04±1.63 ^aa^	13.63±1.21
CMC-Na	30	15.79±0.78	12.88±1.26	12.83±1.25
Aspirin	20	15.88±0.31	11.75±1.75	11.75±1.54
Low-Dose AEE	18	15.42±0.56	9.46±1.54 ^aa^	9.63±1.79^bb^
Medium-Dose AEE	36	15.25±0.63	8.83±1.16^aa^	8.96±0.93 ^bb^
High-Dose AEE	72	15.50±0.71	9.00±1.76^aa^	9.04±1.70 ^bb^
Integration	20+18	15.25±0.50	13.46±0.72	13.50±0.64
Eugenol	18	15.41±0.47	11.25±1.22 ^aa^	11.25±1.52 ^bb^

^aa^ *P*< 0.01 significant difference from CMC-Na group at 24 hours.

^bb^ *P*< 0.01 significant difference from CMC-Na at 48 hours. Integration: integration of aspirin and eugenol (molar ratio 1:1).

**Fig 1:**
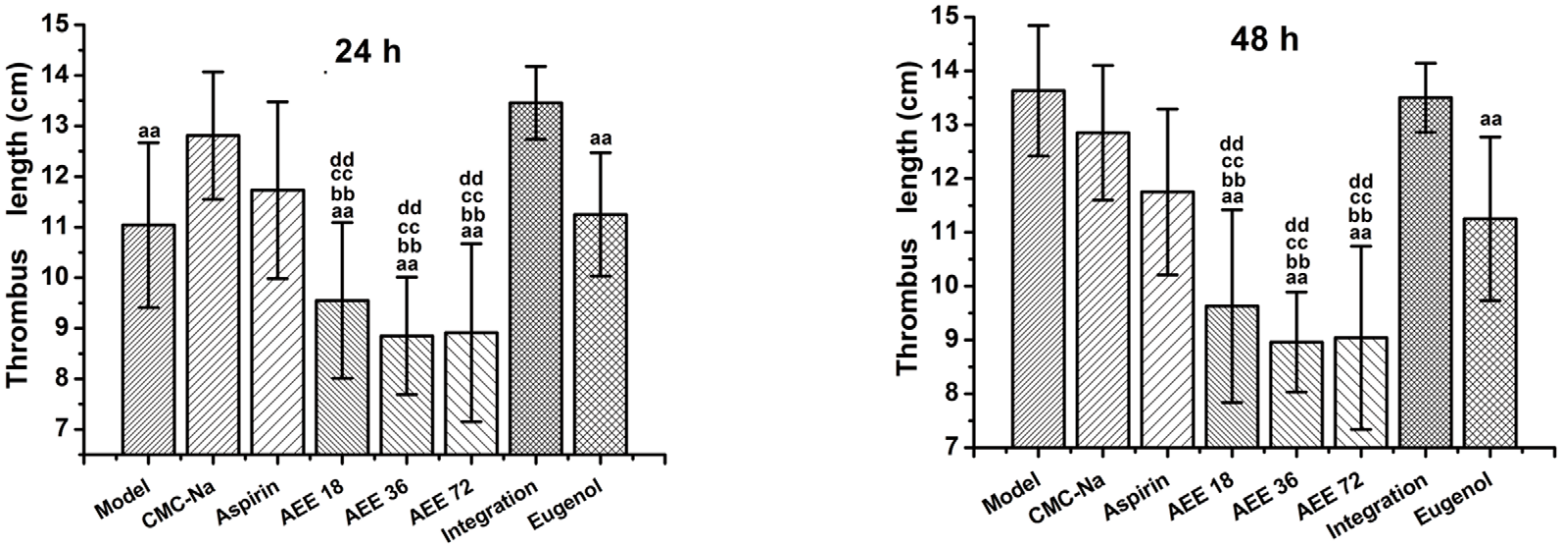
Preventive effects of aspirin, eugenol and AEE on κ-carrageenan-induced rat tail thrombosis length at 24 and 48 h. Each rat was intraperitoneally injected with 20 mg/kg κ-carrageenan dissolved in physiological saline after seven days drug administration, and then the lengths of thrombosis were measured at 24 and 48 hours. Integration: integration of aspirin and eugenol (molar ratio 1:1). All groups were compared with CMC-Na group and three AEE groups were compared with its precursor, aspirin, eugenol and integration groups. ^aa^ *P*<0.01, ^a^ *P*<0.05, significant difference from CMC-Na group. ^bb^ *P*<0.01, ^b^ *P*<0.05, significant difference from aspirin group. ^cc^ *P*<0.01, ^c^ *P*<0.05, significant difference from eugenol group. ^dd^ *P*<0.01, ^d^ *P*<0.05, significant difference from integration group.

**Fig 2:**
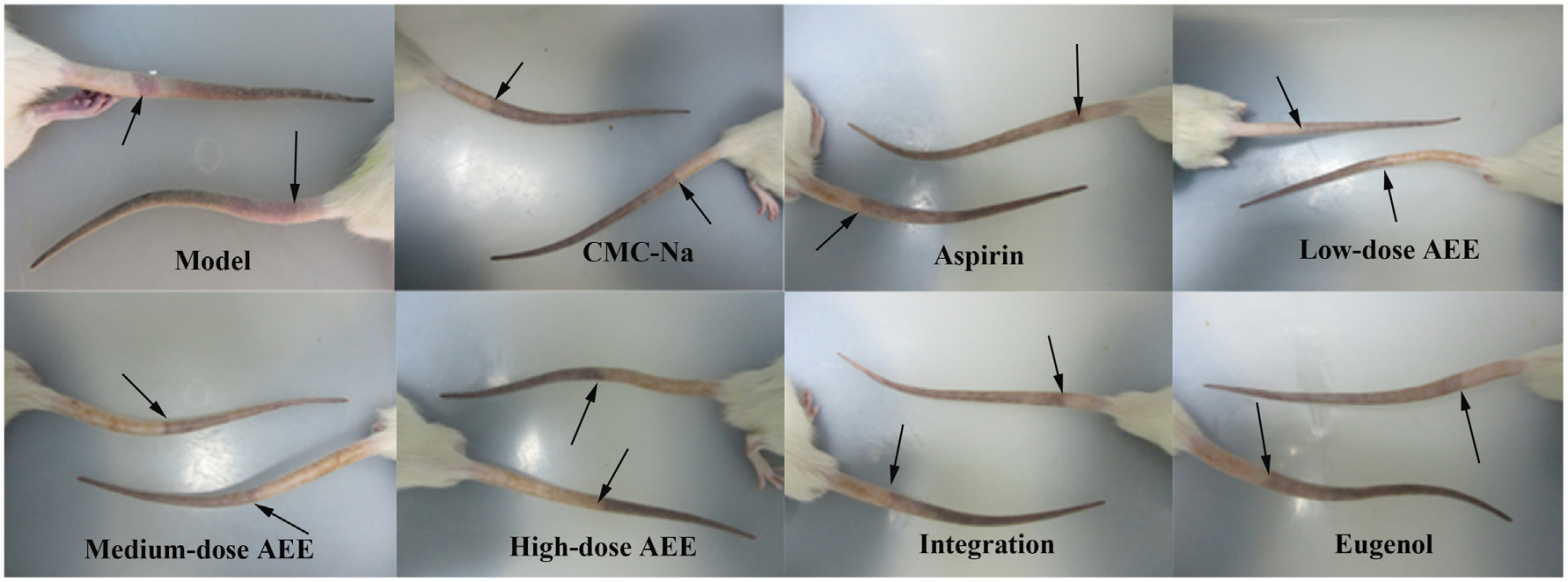
Preventive effects of aspirin, eugenol and AEE on rat tail thrombus induced by κ-carrageenan at 24 hours. Date represents two of ten animals for each group. Swelling and redness was pointed out by arrow. Integration: integration of aspirin and eugenol (molar ratio 1:1).

The results showed that AEE could inhibit significantly thrombus formation in κ-carrageenan-induced thrombosis model in rats (seen in Fig 1). The representative thrombus figures of each group at 24 hours were shown in Fig 2. The average thrombus length in model group was 13.63 cm at 48 hours, whereas the average lengths of thrombus in three AEE groups were 9.63, 8.96 and 9.04 cm at 48 hours. From the results, when compared with the aspirin and eugenol groups, AEE could significantly reduce the thrombus length, which demonstrated that there was a significant difference between AEE and its precursor. Interestingly, there was no significant difference between integration of aspirin and eugenol (molar ratio 1:1) group and model group. Therefore, integration of aspirin and eugenol had no antithrombotic effect, even aspirin and eugenol as two precursors of AEE. The results *in vivo* suggested that AEE can prevent tail thrombosis formation induced by κ-carrageenan.

### Effects of AEE on Coagulation parameters

**Fig 3:**
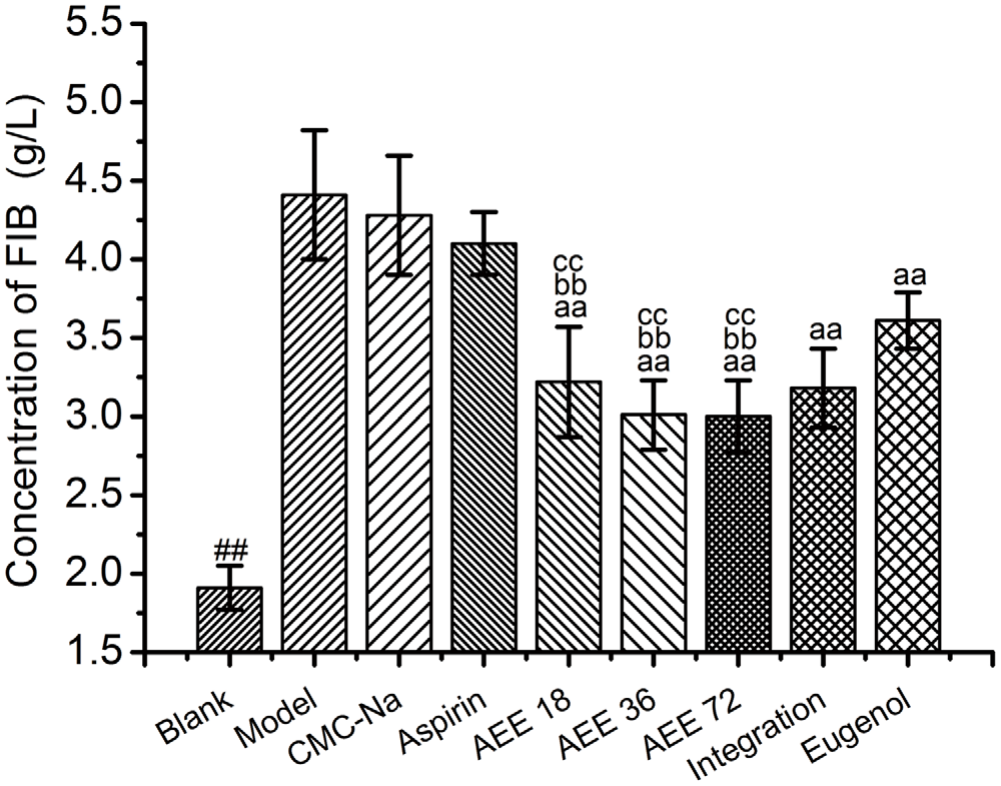
Decreasing effect of AEE on the concentration of FIB in κ-carrageenan-induced rat tail thrombosis model (n = 10). After the last measurement of thrombosis length, serum samples were got for coagulation parameters examination. FIB: fibrinogen. Integration: integration of aspirin and eugenol (molar ratio 1:1). ^##^ *P*< 0.01, ^#^ *P*< 0.05 significant difference from model group. ^aa^ *P*<0.01, ^a^ *P*< 0.05, significant difference from CMC-Na group. ^bb^ *P*< 0.01, ^b^ *P*< 0.05, significant difference from aspirin group. ^cc^ *P*< 0.01, ^c^ *P*< 0.05, significant difference from eugenol group.

Anticoagulant activity of AEE was evaluated by the classical coagulation assays prothrombin time (PT), thrombin time (TT) and fibrinogen (FIB). When compared κ-carrageenan-induced tail thrombosis model group with blank group, the results showed that PT and FIB values increased and TT values reduced significantly. Compared with CMC-Na group, the PT values in aspirin, medium- and high-dose AEE groups were decreased significantly (*P*< 0.01) (seen in Table 2). For TT values, the results in aspirin, eugenol, low- and medium-dose AEE groups were similar to the CMC-Na group. However, the TT values in high-dose AEE group and integration of aspirin and eugenol (ratio 1:1) group were decreased in comparison with CMC-Na group. In addition, the PT and TT values in AEE groups were dose-independent. The concentration values of FIB in eugenol, AEE and integration groups were significantly decreased in comparison with model group (seen in Fig 3).

**Table 2:** Effects of AEE on TT, PT and FIB in κ-carrageenan-induced rat tail thrombosis (n = 10). Note: 2 ml blood samples in vacuum tube were diluted by 3.2% sodium citrate on the proportion of 9:1, then serum be got after centrifuging for 15 min in 3000 rpm for blood coagulation parameters examination. Values are presented as mean ± SD (n = 10).

Group	Dose(mg/kg)	PT(s)	TT(s)	FIB(g/L)
Blank	—	8.51±0.24^##^	42.51±1.6^##^	1.91±0.14^##^
Model	—	9.14±0.17	37.04±1.33	4.41±0.41
CMC-Na	30	9.06±0.24	37.95±1.11	4.28±0.38
Aspirin	20	8.65±0.26**	38.38±1.32	4.10±0.20
Low-dose AEE	18	9.07±0.44	36.78±1.46	3.22±0.35**
Medium-dose AEE	36	8.45±0.28**	38.72±0.96	3.01±0.22**
High-dose	72	8.65±0.38**	35.45±1.50**	3.00±0.23**
Integration	20+18	8.84±0.24	35.58±0.87**	3.18±0.25**
Eugenol	18	9.28±0.32	37.07±0.83	3.61±0.18**

^##^ *P*< 0.01 significant difference from model group.

**P< 0.01, significant difference from CMC-Na group. PT: prothrombin time, TT: thrombin time, FIB: fibrinogen. Integration: integration of aspirin and eugenol (molar ratio 1:1).

Prolongation of TT suggests the inhibition of thrombin activity or fibrin polymerization. From the experimental results, there was no statistical difference in drug administration group except for high-dose AEE and integration group. TT values in high-dose AEE and integration group were shorter than those in CMC-Na group. When the body sustained thrombotic disease, TT value should become short. The thrombosis formation in rat tail and the change of FIB concentration may be the reasons for the decrease of TT value.

Prolongation of PT demonstrates the inhibition of the extrinsic pathway of coagulation. The PT values in model group were prolonged in comparison with blank group, which is very interesting. This result may be caused by the thrombosis formation which consumed the coagulation factors. Coagulation factors play a key role in activating prothrombin and the lack of coagulation factors can lead the prolongation of PT value. AEE decreased the PT values through the inhibitation of thrombosis formation. The decrease of FIB concentration in AEE group is helpful to inhibit the conversion of fibrinogen to fibrin. Therefore, AEE possessed better effect on thrombosis prevention from the concentration reduction of FIB.

### Anticoagulant effect of AEE *in vitro*

**Fig 4:**
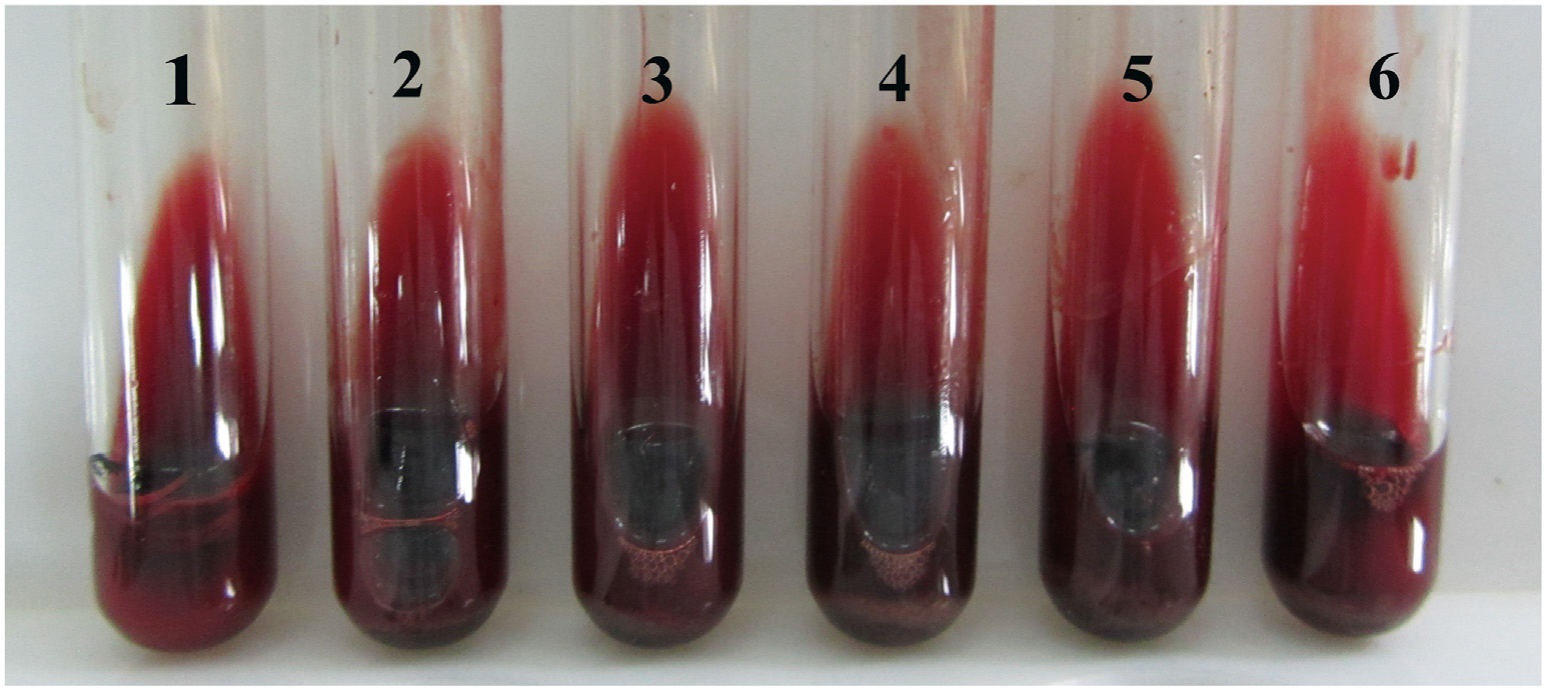
Drug effect of AEE with different concentrations on fresh rat blood *in vitro*. The bloods were coagulated and formed dark red blood clots at the bottom of the tubes, which indicated that AEE had no notable effect on clot of rat blood. 1: normal fresh rat blood; 2: rat blood with 200 μL DMSO; 3: rat blood with 1 μg AEE in 200 μL DMSO; 4: rat blood with 10 μg AEE in 200 μL DMSO; 5: rat blood with 100 μg AEE in 200 μL DMSO; 6: rat blood with 200 μg AEE in 200 μL DMSO.

The anticoagulant effects of AEE on fresh rat blood were tested. However, the results showed that AEE had no notable effect on clotting of rat blood, even the concentration of AEE at 200 μg/ml. The bloods were coagulated and formed dark red blood clots at the bottom of the tubes (seen in Fig 4). There was no difference between the control and drug treatment groups. Unlike heparin sodium or EDTA-K_2_, these results indicated that AEE might have different mechanism for inhibiting thrombosis formation.

### Effects of AEE on platelet aggregation *in vitro*

**Fig 5:**
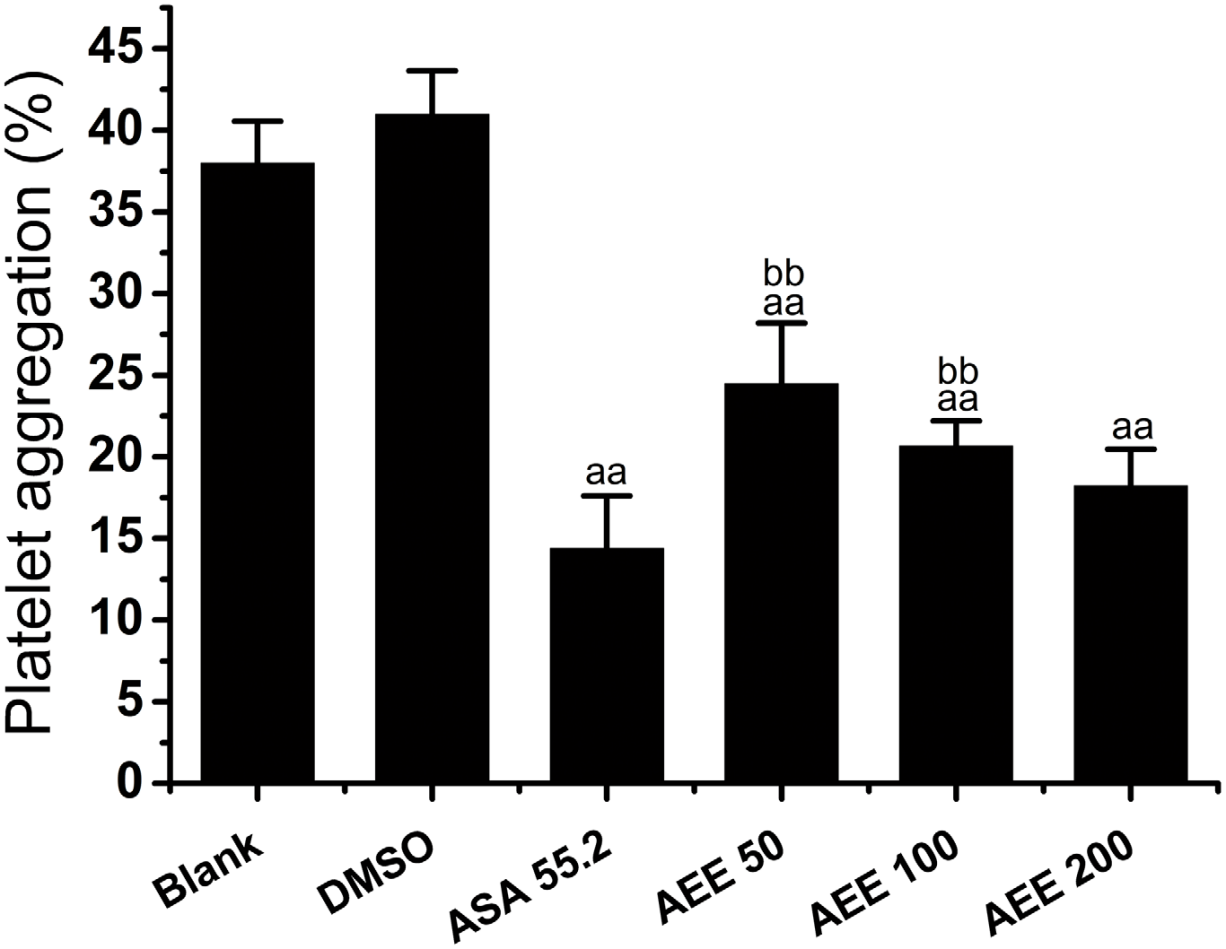
Effect of AEE on platelet aggregation *in vitro*. After incubation of rat platelets with AEE at concentrations ranging from 50 μg/ml to 200 μg/ml for 10 minutes, the platelet aggregation activities was assessed using ADP (5 μM). Data are expressed as mean ± SD (n = 8). ^aa^ *P*< 0.01, ^a^ *P*< 0.05, significant difference from DMSO group. ^bb^ *P*< 0.01, ^b^ *P*< 0.05, significant difference from aspirin group. DMSO: dimethyl sulphoxide; ASA: acetyl salicylic acid (aspirin).

To assess the effect of AEE on platelet aggregation, classical light transmission aggregometry was performed after incubation of rat platelets with AEE at different concentrations ranging from 50 μg/ml to 200 μg/ml. As shown in Fig 5, compared with normal group, DMSO appears no significant effect on the platelet aggregation. The results of DMSO on platelet aggregation in this experiment were similar as the previous study [29]. After incubation for 10 minutes, AEE significantly inhibited platelet aggregation in comparison with normal control group. Platelet aggregation rates were approximately 24.5% at 50 μg/ml, 20.6% at 100 μg/ml and 18.2% at 200 μg/ml, respectively, which showed that AEE dose-dependently inhibited ADP-induced platelet aggregation. In the experiment, aspirin was used as positive control drug. The results showed that the inhibitory effect of AEE was less potent at concentrations of 100 μg/ml in comparison to equimolar doses of aspirin.

### Hematology

In the hematological analysis (seen in Table 3), leukocyte count (WBC) and monocyte count (MONON) in model group were significantly increased compared with the blank group, but platelets (PLT) was sharply reduced. No other changes were observed between blank and model groups. The increase of WBC and MONON may be caused by inflammation which was induced by κ-carrageenan. The formation of tail thrombosis could deplete circulating PLT to result in the sharp reduction of PLT.

**Table 3:** Hematological findings in rats administrated intragastrically with AEE. Note: After the last measurement of thrombosis length, two ml blood samples were collected into EDTA-K_2_ vacuum tubes for hematological analysis.

Variable	Unit	Blank	Model	CMC-Na	Aspirin	Low AEE	Mid AEE	High AEE	Integration	Eugenol
WBC	10^^^9/L	5.38±0.98^##^	7.68±1.64	7.17±1.78	8.32±1.6	7.38±2.09	7.95±1.92	7.89±1.36	7.04±1.40	6.97±1.32
RBC	10^^^12/L	8.14±0.45	7.80±0.45	7.69±0.40	7.86±0.32	7.20±0.50**	7.04±0.41**	7.29±0.49*	7.12±0.33**	7.53±0.34
HGB	g/L	153.7±7.6	148.8±9	142.4±7.8	149±6.2*	136±8.7*	129±6.9**	134.8±8.8*	132.4±5.1**	141.1±6
HCT	%	46.1±1.6	44.0±2.5	43.6±2.4	43.4±1.5	41.1±1.6**	40±1.5**	41.7±2.6*	40.9±1.6**	43.0±1.6
MCV	fL	56.64±1.46	56.47±0.5	56.54±0.84	55.23±0.98	57.02±1.24	56.02±1.01	57.14±1.30	57.47±0.90	57.19±1.22
MCH	pg	18.88±0.26	19.08±0.24	18.48±0.26	18.95±0.24	18.92±0.24	18.35±0.31	18.49±0.21	18.47±0.26	18.72±0.26
MCHC	g/L	333.44±7.09	337.91±2.3	326.75±3.42	343.15±5.3**	332.77±7.95	322.23±6.64	323.64±6.54	321.79±5.13	329.08±2.84
PLT	10^^^9/L	851±68^##^	703±49	782±49	681±55**	641±80**	675±84**	680±44**	602±41**	745±38
MONON	10^^^9/L	0.11±0.08^##^	0.53±0.16	0.59±0.24	0.59±0.14	0.58±0.19	0.60±0.16	0.59±0.10	0.55±0.19	0.64±0.17
RDW-CV	%	14.91±0.54	14.34±0.43	14.11±0.29	14.26±0.67	14.25±0.66	14.75±0.59	14.23±0.50	14.66±0.47	14.21±0.48
PDW	%	9.48±0.29	9.31±0.27	9.46±0.44	9.01±0.35	9.54±0.54	9.51±0.38	9.65±0.41	9.27±0.42	9.69±0.35
MPV	fL	8.43±0.14	8.29±0.12	8.37±0.18	8.2±0.16	8.52±0.22	8.4±0.23	8.64±0.26	8.35±0.3	8.59±0.22
RDW-SD	%	29.09±1.81	28.53±0.86	28.05±0.68	27.57±1.6	28.45±1.12	29.96±1.38	28.39±0.93	29.59±0.85	28.56±1.35

^##^ *P*< 0.01,

^#^ *P*< 0.05 significant difference from model group.

**P<0.01,

*P< 0.05Significant difference compared with CMC-Na group. Integration: integration of aspirin and eugenol (molar ratio 1:1).

When compared with the CMC-Na group, the value of red blood cell (RBC), hemoglobin (HGB) and hematocrit (HCT) were significantly reduced in AEE groups. In regard to RBC, aspirin and eugenol had no effect on the reduction of RBC. However, the results in AEE and integration groups showed significant difference in comparison with aspirin and eugenol. The results suggested that AEE and integration of aspirin and eugenol can reduce RBC value in κ-carrageenan induced thrombosis model. The results of HGB and HCT were similar to RBC owing to the close relationship among HGB, RBC and HCT. With the RBC reduced, the values of HCT, and HGB were reduced correspondingly. Therefore, AEE may have a positive effect on blood viscosity through the reduction of HCT value and FIB concentration. HCT is associated with blood viscosity and blood flow as one of crucial factor. In the experiment, the reduction of HCT values in AEE groups may be caused by the reduction of RBC. Based on the experimental results, it is essential to investigate the effect of AEE on blood viscosity parameters in the further study.

PLT count was decreased in AEE group when compared with CMC-Na group. There was no statistical difference in eugenol group, which may suggest eugenol made no influence on PLT. The values in AEE groups showed significant difference in comparison with eugenol group. Notably, PLT value in integration group was the lowest in all groups. The mean PLT values in AEE groups were also lower than aspirin and eugenol groups. The influence of AEE on PLT in κ-carrageenan induces thrombosis model was similar with the results in 15-day oral dose toxicity study [14]. The reduction of PLT values in AEE group may also make contribution on the inhibition of thrombosis formation and reduction of blood viscosity.

## Discussion

Based on the design of prodrug principal, AEE is confirmed to be effective against the symptoms of inflammation, fever and soreness [17–18]. The previous study showed that this compound could reduce significantly the value of TG, TC and platelet counts at the dosage of 50 mg/kg [14], which is very beneficial to the therapy of atherosclerosis and thrombosis. Meanwhile, there were no histopathological changes in any organ from the rats given AEE at the dose of 50 mg/kg. This result suggested that AEE was a safe drug with relatively less gastrointestinal (GI) toxicity. Based on this fact, it is essential to evaluate the preventive effect of AEE in animal thrombosis model.

Carrageenan is straight-chained sulfur-containing macromolecular polysaccharides that are composed of repeating units of D-galactose and 3, 6-anhydro-D-galactose. Kappa-carrageenan is widely used to induce tissue inflammation and tail thrombosis in different animals such as mouse and rat. According to the pathological studies, κ-carrageenan can cause local blood vessel inflammation and endothelial cell injury through releasing inflammation factors, which may lead to the formation of thrombus [30]. Inflammation, especially in blood vessel, is considered to make contribution to thrombosis. On the contrary, blood clots can cause inflammation [31]. In our experiment, the increase in leukocyte count and monocyte count could prove that κ-carrageenan cause acute and systemic inflammation in model group. However, there was no significant difference for two indexes between drug treatment groups and model group. These results may be from the thrombosis which kept continuous appearance of inflammation and led two indexes to be in higher status.

Thrombosis starts with adhesion of PLT to the vessel wall, followed by aggregation of additional PLT [32–33]. PLT reactions are also strong stimulus to the necessary clotting factors [34]. Therefore, PLT play a key role in the formation of thrombosis. As cyclooxygenase inhibitors, aspirin is used therapeutically in the prevention of cardiovascular disease [35]. Aspirin inhibit PLT aggregation through blocking TXA_2_ synthesis which is a potent inducer of PLT aggregation and causes vasoconstriction [36–37]. In our previous study, the metabolites of AEE *in vivo* and *in vitro* have been confirmed [16]. In total, five metabolites were detected, including salicylic acid, salicylic acid glucuronide, salicylic acid glycine, eugenol glucuronide and eugenol sulfate. Salicylic acid, which is also the same metabolite of aspirin, is found as one of the major metabolites. It is deduced that AEE has similar effects like aspirin in preventing cardiovascular disease. So the effects of AEE ensued not from AEE itself but from salicylic acid and eugenol decomposed by the enzymes after absorption, which showed their original activities again and acted synergistically. 15-day oral dose toxicity study showed that AEE at the dose of 50 mg/kg is able to reduce significantly the number of PLT [14]. In hematological analysis under the experiment, the index of PLT count was significant different between administration groups and CMC-Na group. The results showed that AEE had positive effects on PLT reduction, and the action of AEE on PLT reduction was stronger than aspirin and eugenol. Before κ-carrageenan was administrated, PLT may partly reduced by AEE. The different length thromboses deplete residual available PLT after κ-carrageenan administrated. This may be one of the reasons to explain partly why shorter thrombotic length appeared in AEE groups and longer thrombotic length appeared in model and other groups.

Thrombin is at the center of the process of blood clotting [38–39]. In order to control thrombin and regulate blood clotting carefully, thrombin is built as an inactive precursor, prothrombin [40]. When the tissue is cut, the blood flows out of the blood vessels and encounters tissue factor. After that, tissue factor activates a few molecules of Factor VII. These then activate a lot of Factor X. Finally, these activate even more thrombin. Thrombin catalyzes conversion of fibrinogen to fibrin to assemble into large stringy networks [41]. These networks then trap lots of blood cells to form the dark red scab. Under the present experiment, PT was prolonged in model group and shortened in AEE groups. AEE inhibited the thrombosis formation, and then improved the level of coagulation factors and reduced PT values. For FIB, AEE reduced significantly its concentration in rat bloods. The reduction of FIB inhibited the network of fibers which is essential for thrombosis formation. TT in model group was shorter than in blank group, which may be caused by FIB increase. TT values were shorter in high-dose AEE group in comparison with CMC-Na group, which suggested that high-dose AEE may had a negative impact on inhibition of thrombin activity or fibrin polymerization. The reasons for the difference of TT value in high-dose AEE and integration group were needed to be investigated in the further study.

In this study, the antiplatelet and anticoagulant effects of AEE *in vitro* were examined. The effects of AEE on platelet function was determined by measuring ADP-induced platelet aggregation. The results showed that AEE produced obviously antiplatelet effect on ADP-induced platelet aggregation. The inhibitory effect of AEE was less potent in comparison with aspirin at equimolar dose, which indicated that AEE might have different mechanism for inhibiting thrombosis formation. Meanwhile, AEE showed no anticoagulant effect on fresh rat blood. This result may suggest that metabolic processes in the body is necessary for the drug effect of AEE.

After strict biological and toxicological research and test, pure CMC had been approved as food additive by WHO and FAO [42]. In order to eliminate the effect of CMC-Na, CMC-Na was also administrated as control group. Therefore, the antithrombotic effect of AEE is not related to CMC-Na. To be notice, the average lengths in CMC-Na group at 24 hours was longer than model group and platelet count was higher than other groups in hematological analysis. These results may indicate that CMC-Na has negative influence on antithrombosis at 24 hours but no influence at 48 hours. Tween 80 is widely used as emulsifier in food industry and drug production. In general, the body has a great tolerance to Tween 80 [43]. Because the amount of Tween 80 was fewer, the biological preventive effect of Tween 80 on thrombosis in this experiment is not investigated.

From the total results of the experiment, 36 mg/kg as medium-dose AEE may be approved for antithrombotic use in rat tail thrombosis model induced by κ-carrageenan. The average thrombosis length in medium-dose AEE group was shorter than that in low-dose AEE group. Meanwhile, there was no statistical difference in the thrombosis length between medium and high dose AEE groups. Moreover, there was no difference between medium-dose and high-dose AEE groups on FIB and PLT reduction.

All in all, AEE displayed strong preventive effect on thrombosis in κ-carrageenan-induced rat tail thrombosis model and showed better antithrombotic effect than its precursor, which indicated that AEE could be a good candidate for antithrombotic agent. From the experiment results, the preventive effect of AEE on thrombosis may come from the reduction of FIB concentration, PLT, inhibition of platelet aggregation and the change of PT and TT values. Certainly, more studies such as evaluation for inflammation biomarker, platelet aggregation, blood stream changes and coagulation factors are needed to characterize AEE and to investigate its action mechanism.
